# Beef Tea and Meat Preparations

**Published:** 1902-02-15

**Authors:** Marcus R. P. Dorman


### The Institutional Workshop.

### BEEF TEA AND MEAT PREPARATIONS.

By Marcus R. P. Dorman, M.D.

### IV.—THE ECONOMY OF MEAT EXTRACTS AND PREPARATIONS.

[In Chapter II. of this series, which appeared in our issue of February 1st, there is a printer's error to which we wish to draw attention. By the substitution of figure 3 for figure 8 the total dry solids contained in Lemco were given as 32·2 instead of 82·2 per cent. By this error Lemco was made to appear the weakest instead of, as it should have been, the strongest of the three preparations mentioned.]

It is admitted by all that in the treatment of the sick the best of everything should be used, and it is therefore far more important to demonstrate the advantages of using meat extracts and preparations rather than beef tea from a chemical and clinical standpoint than to point out that the former are much cheaper. It is, however, very necessary to conduct hospitals and other institutions on strictly economical principles; and if it can be shown that the present method of making beef tea is more costly than using commercial products and is not counterbalanced by a single scientific advantage, then no reason remains why the domestic solution should not be largely or entirely replaced by the manufactured article. We have pointed out that beef tea is very variable in its composition, so that it is practically impossible to know what amount of extractives the patient is swallowing with the quantity made for him, whereas we can estimate almost to a grain the amount of extract and proteids in the solutions made from commercial preparations. Next it must be clearly borne in mind that neither beef-tea nor pure extracts can be compared in efficacy to meat preparations in those cases requiring nourishment as well as stimulants. In comparing the relative cost, many items, such as the fuel, labour, and time incidental to the preparation of beef-tea are, perhaps, impossible to estimate with any degree of accuracy, but they must total to a considerable sum in the course of time. In order to simplify the question we will, however, omit this item. As we have shown, our knowledge of the physiological action of food is not sufficient to enable us to reduce its value into terms of benefit units with mathematical accuracy, but since the composition of beef-tea and meat extracts is for all practical purposes similar, we are, therefore, justified in basing our comparison simply on the cost of meat. It has been estimated that a hospital with a daily average of about 300 beds will consume something like 30,000 pints of beef-tea in a year, and that the cost of this with meat at 4d. and 6d. per pound, allowing one pound to the pint, amounts to £500 and £750 respectively.[1] It is easy to see that, working on the estimate given in Article II., the cost of 30,000 pints of a solution of Lemco, for example, would be less than half the latter sum.[2] This calculation merely refers to the amount of solid extract given, and the same formula cannot therefore be applied to the nourishing preparations. In order to approach to an accurate idea of their money value we should have not only to estimate the amount of extractives, but also the cost of the lean meat which would give an equivalent in nourishing properties. I have no intention of attempting to give actual figures, but, roughly speaking, such preparations as Oxo and Invalid Bovril seem to me at the price sold to possess three or four times the stimulant and dietetic value of an equal expenditure on beef-tea and lean meat at London prices. Indeed there can be no doubt that all the preparations mentioned in these articles, value for value, in stimulant and dietetic properties, are actually much cheaper than beef-tea or meat itself when bought from an English butcher. Nor is it difficult to discover how it is that commercial companies with large capitals on which to pay dividends and who also spend huge sums on advertising can still afford to manufacture preparations of great excellence and sell them at a less price than that asked for plain meat. The Liebig Company possess extensive tracts of country near Fray Bentos, Uruguay, where their cattle are bred and reared and where their chief factory is situated. The Bovril Company purchase their extracts from Queensland, New South Wales, and Canada, the process in the London factory consisting only in blending them and adding the "beef meal." The Armour Laboratory is situated at Chicago, the city of stock yards. Prime English beef in a shop in London costs 10d. or 11d. per lb., the finest beef costs to these companies about one-tenth of that sum. It is therefore easy to understand that, even if it requires 40 lb. of lean beef to make 1 lb. of extract, the present prices leave a handsome margin for working expenses and profit.

The chief points to bear in mind in estimating the monetary value of any of these preparations are (1) the amount of water they contain; (2) the total solid extracts as compared with the average beef-tea, and (3) the nutritive value in terms of lean meat. There is, however, another important advantage in using meat preparations, which it is impossible to estimate mathematically, and that is the effect of feeding a patient on a diet which he relishes, enjoys, and can digest. Fever patients are usually treated with large quantities of milk, which in many cases is not digested at all but passes through the intestines in a curdled condition, giving rise to the most troublesome and painful flatulence. Again, hysterical subjects and neurasthenics are, often with difficulty, induced to swallow pints of this food, and undoubtedly are eventually fattened, but it is extremely doubtful whether their mental condition improves as rapidly as their weight. In cases of phthisis also the digestive system is frequently not in the condition to deal successfully with the amount of ordinary food required to sustain the body vitality. The preparations containing lean meat or myosin albumin are particularly suitable for all such, and can be substituted with advantage for part of the milk diet. Many can be given either in the shape of a warm or cold solution, some with aerated water as a pleasant refreshing drink, others in the form of jelly or as sandwiches, or to form the basis of many appetising dishes. With a little thought it is, indeed, possible to devise a great variety of pleasant and digestible substitutes for the cold clammy milk and soda water, and the monotonous domestic beef tea. We have many excellent preparations to hand, and it only remains to ascertain and utilise their various properties in suitable cases.

[1] Hospital Expenditure: The Commissariat, p. 102.

[2] The actual figure, allowing ⅝ oz. of Lemco to the pint at 5s. 6d. per lb., is £322.

